# Single-port sleeve gastrectomy: a comparison between transumbilical and left hypochondrium approaches

**DOI:** 10.1007/s00464-025-11529-3

**Published:** 2025-02-10

**Authors:** Hussein Abdallah, Joseph Derienne, Rodi Courie, Cosmin Sebastian Voican, Gabriel Perlemuter, Gianfranco Donatelli, Ibrahim Dagher, Hadrien Tranchart

**Affiliations:** 1 Department of Minimally Invasive Digestive Surgery, Antoine Béclère Hospital, AP-HP, 157 Rue de La Porte de Trivaux, 92141 Clamart, France; 2 Paris-Saclay University, 91405 Orsay, France; 3 Department of Hepato-Gastroenterology and Nutrition, Antoine Béclère Hospital, AP-HP, 92140 Clamart, France; 4 Interventional Endoscopy Unit, Private Hospital Des Peupliers-Ramsay Santé, 75013 Paris, France

**Keywords:** Bariatric, Obesity, SILS, Single-port, Umbilical, Sleeve gastrectomy

## Abstract

**Background:**

Left hypochondrium (LHC) approach has been routinely used in our department for performing single-port sleeve gastrectomy (SPSG). Starting from 2019, a transumbilical approach (TU) has been adopted in selected patients. The aim of this study was to report and compare our results of both approaches (LHC and TU) with special focus on incisional hernia (IH).

**Methods:**

The data of patients who underwent sleeve gastrectomy via both approaches between 2019 and 2022 were retrospectively analyzed. An assessment of IH rate was carried out by reviewing abdominal computed tomography scans performed one year after surgery.

**Results:**

During the study period, 449 patients who underwent SPSG were included in the final analyze. Patients in the TU group (*n* = 136, 30%) were more frequently female with a lower BMI and fewer comorbidities. An umbilical hernia was observed in 60% of patients in the TU group. Operative duration was longer in the LHC group (80 min vs. 64 min, *P* < 0.0001). Early complications rates did not differ between the groups (1.9% LHC vs. 0.7% TU, *P* = 0.353). During follow-up, 65 patients (14%) developed an IH: 9.9% and 25% in the LHC and TU groups, respectively (*P* < 0.0001). Weight loss and comorbidities resolution at 1 year were globally similar between the two groups.

**Conclusion:**

We have demonstrated the feasibility, safety, and efficacy of SPSG via both LHC and TU approaches. The advantage of the LHC approach is its routine applicability. The TU approach offers an esthetic advantage and a shorter operative time but is associated with a much higher IH rate.

**Graphical abstract:**

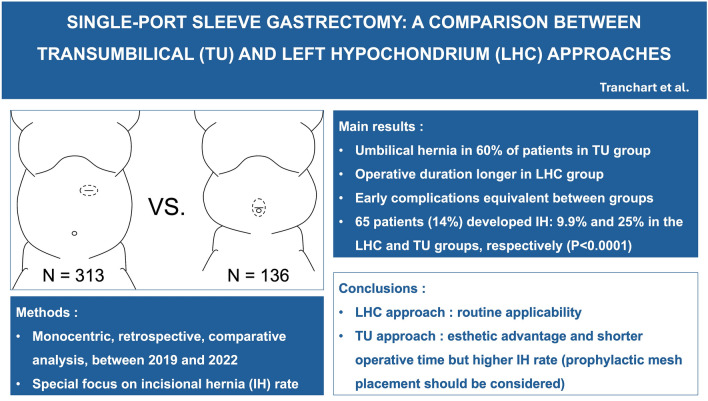

Obesity is an alarmingly increasing global public health challenge and a major determinant of disability and death. In 2022, more than a 1 billion children, adolescents, and adults worldwide had obesity [1].

Laparoscopic Sleeve Gastrectomy (LSG) is the most adopted procedure by bariatric surgeons worldwide [2] and its efficacity has been confirmed [3]. Traditionally LSG is done by performing three to five skin incisions for trocars placement. In recent years, many teams have tried to reduce the aggressiveness of this surgery by developing minimally invasive techniques that could extend the advantages of laparoscopic surgery.

Single-port laparoscopic sleeve gastrectomy (SPSG) was first described in 2008 by Saber et al. [4]. Since then, numerous studies have demonstrated the safety and feasibility of this procedure [5–11]. However, single-incision laparoscopic surgery (SILS) is associated with a paradoxically increased risk of incisional hernia (IH) [12] and the adoption of this approach in bariatric centers remains a subject of debate.

A standardized and reproducible technique of SPSG was designed in our department using specific instruments [5]. Since 2010, this approach has been routinely used in all patients and more than 4000 procedures have been performed. At the beginning of our experience, intraabdominal access was systemically obtained through a left hypochondrium (LHC) incision. This approach has been standardized and is feasible in all patients with results equivalent to conventional approach. In 2015 we started to perform this procedure by using the transumbilical (TU) access and by 2019 it became our chosen approach in selected patients.

The purpose of our study was to retrospectively analyze and describe our experience of SPSG on a prospectively collected data and to compare the outcomes between its two approaches (TU and LHC) with special focus on IH.

## Materials and methods

Data from all consecutive patients who underwent SPSG in our bariatric institution between January 2019 and June 2022 were prospectively collected (after informed consent by patients and institutional review board approval) and retrospectively analyzed. This study was performed according to the Strengthening the Reporting of Observational Studies in Epidemiology (Strobe) guidelines. Operative and postoperative outcomes were analyzed in patients with more than 1-year follow-up.

All patients underwent a meticulous preoperative examination by a multidisciplinary team as previously described [5]. Patients were eligible for surgery if they had a body mass index (BMI) of 40 kg/m^2^ or higher, or a BMI between 35 and 40 kg/m^2^ with significant comorbidities according to French guidelines [13].

A TU approach was performed under two conditions: if the left quadrant incision site was less than 5 cm from the umbilicus in patients with a BMI < 50 kg/m^2^.

### Surgical technique

The surgical technique for performing a SPSG via the LHC and the TU has already been described in detail [14]. Briefly, for LHC approach, the multiport is introduced through a 2–3 cm transverse incision, 8 cm below the costal margin and 2–3 cm to the left of the midline. For TU approach, a 2–3 cm vertical upper umbilical incision is used.

A specific instrumentation is required including a multiport single-access device, a 10-mm flexible tip laparoscope or a standard 10-mm rigid 30° laparoscope, a double-curved grasper and a thermofusion device [14]. Transection of the stomach is done using a 60-mm endoscopic stapler. All cartridges are buttressed with bioabsorbable staple-line reinforcement. The use of long instruments (thermofusion and stapler) is imperative for TU approach.

The specimen is removed through the single-port’s protective skirt. Closure of the two rectus abdominis fascia for the LHC approach and linea alba for TU approach is performed using running sutures of an absorbable braided suture size 1.

### Postoperative management and follow-up

Patients were allowed free liquid intake and placed on a semiliquid diet 2 days after surgery and solid intake was allowed gradually during the first 3 weeks after operation.

Patients took part in a standard follow-up bariatric program including 4 visits to the outpatient clinic during the first postoperative year, with clinical examination of the surgical wound and of the parietal wall. Systematic computed tomography (CT) scan without contrast injection was performed in all patients one year after surgery.

### Data collection and statistical analysis

Demographic data were obtained from a prospective electronic database. Data recorded included patients’ characteristics, perioperative course, and follow-up details. Complication severity was stratified according to the modified Clavien classification. If a patient had two or more complications, the most severe was taken in account. Complications and operative mortality considered were those that occurred within 30 days of surgery, or at any time during the postoperative hospital stay. Staple-line leakage was regarded as a complication when an intraabdominal abscess, requiring drainage or antibiotic treatment, was found on CT scan or during re-laparoscopy. Several outcomes such as weight loss and remission of comorbidities were evaluated at 12 months of follow-up. Discontinuation of all medication for the treatment of a co-morbidity (or end of continuous positive airway pressure use) was regarded as remission.

Percentage excess weight loss (EWL) was calculated using the following formula: %EWL = [weight loss (kg)/excess weight (kg)] × 100. Excess weight was based on the patient’s ideal weight, with a BMI of 25 kg/m^2^. The assessment of IH was done clinically and radiologically.

Statistical analyses were performed with GraphPad Prism 5.0 (GraphPad Software, Inc., San Diego, CA). The analyses were checked and validated by a statistical expert. Continuous variables are described as medians and interquartile ranges or means and standard deviations, according to their distribution. Categorical variables are described as absolute numbers and percentages. The two-tailed unpaired t-test or the Mann–Whitney test was used to compare continuous variables and the Chi-square or Fisher’s exact test was used to compare categorical variables, as appropriate. The 5% level was considered as significant.

## Results

Among 514 patients who underwent primary bariatric surgery between January 2019 and June 2022, 65 patients with missing clinical and/or radiological reports were excluded from the study; thus, the total number of patients enrolled in our analysis was 449. A TU approach was used in 136 patients (30%). The mean follow-up time was 24 ;months.

The median age was 40 years [31–49] and the median BMI was 41.4 kg/m^2^ [38.2–45.8]. There were 93 males (20.7%) males and 356 (79.3%) females. The two groups did not differ in age, whereas, as expected, patients in the TU group were more frequently women (92.6%) and had a lower BMI (*P* < 0.0001). Again, as expected, patients in the LHC group had more comorbidities. The demographic and clinical characteristics of the patients are listed in Table 1.

**Table 1:** Baseline characteristics of patients

Characteristic	Overall (*n* = 449)	LHC group (*n* = 313)	TU group (*n* = 136)	*P*
Gender (female/male)	356/93	230/83	126/10	< 0.0001
Age, year, median [IQR]	40 [31–49]	41 [35–48]	40 [34–47]	< 0.006
Weight, kg, median [IQR]	114 [103–128]	118 [104–135]	107 [97–119]	< 0.0001
BMI, kg/m^2^, median [IQR]	41.4 [38.2–45.8]	41.78 [37.4–47.1]	40 [37–43.6]	< 0.0001
Comorbidities				
Diabetes, *n* (%)	38 (8.4)	33 (10.5)	5 (3.7)	0.016
Hypertension, *n* (%)	94 (20.9)	74 (23.6)	20 (14.7)	0.032
Dyslipidemia, *n* (%)	57 (12.7)	48 (15.3)	9 (6.6)	0.010
OSAS, *n* (%)	182 (40.5)	155 (49.5)	27 (19.9)	0.001
Cardiovascular disease, *n* (%)	17 (3.8)	15 (4.8)	2 (1.5)	0.090
Fatty liver disease, *n* (%)	120 (26.7)	82 (26.2)	38 (27.8)	0.701
Tobacco use, *n* (%)	4 (0.8)	4 (1.2)	0	–

*LH* left hypochondrium, *TU* transumbilical, *IQR* interquartile range, *BMI* body mass index, *OSAS* obstructive sleep apnea syndrome

Mean operative time was 75 min [62–95]. Operative time was significantly longer in the LHC group (80 min vs. 64 min, *P* < 0.0001). In both groups, SPSG was performed successfully without requiring additional ports, conversion to laparotomy and without significant (> 100 ml) intraoperative bleeding. Median length of stay was 3 days. Operative and postoperative outcomes of patients in LHC and TU groups are depicted in Table 2.

**Table 2:** Operative and postoperative outcomes

Outcomes	LHC group (*n* = 313)	TU group (*n* = 136)	*P*
Operative time, min, median [IQR]	80 [67–105]	64 [51–76]	< 0.0001
Additional extraport, *n* (%)	0	0	–
Conversion to laparotomy, *n* (%)	0	0	–
Significant intraoperative bleeding (> 100 ml), *n* (%)	0	0	–
Abdominal drainage, *n* (%)	0	0	–
30-day postoperative complications, *n* (%)	6 (1.9)	1 (0.7)	0.353
Bleeding (intraabdominal or intraluminal), *n* (%)	2 (0.4)	1 (0.7)	0.908
&nbsp;Staple-line leak, *n* (%)	4 (0.9)	0	–
Dindo-Clavien Grade ≥ IIIa, *n* (%)	6 (1.9)	1 (0.7)	0.353
Incisional hernia, *n* (%)	31 (9.9)	34 (25)	< 0.0001
Length of stay, day, median [IQR]	3 [3–3]	3 [3–3]	–

*LHC* left hypochondrium, *TU* transumbilical, *IQR* interquartile range

### Early postoperative complications (< 30 days)

Seven early complications occurred in seven patients, resulting in an overall early complication rate of 1.5%. Early complication rates did not differ significantly between groups and were statistically similar despite a minor increase in the LHC group (1.9% vs. 0.7%, *P* = 0.353). Severe complication rates (Dindo–Clavien Grade ≥ IIIa) were also comparable between the groups. No deaths were reported in either group.

There were four staple line leaks (0.9%), all occurring in the LHC group, with none in the TU group. The leaks were detected after postoperative day 4. All patients with leaks were managed and treated endoscopically. Two patients required initial reintervention due to septic shock with hemodynamic instability and were managed laparoscopically by evacuation and drainage of a peri-gastric abscess, followed by subsequent endoscopic drainage.

Three patients (0.6%) experienced significant postoperative bleeding from the staple line, detected by a CT scan performed on postoperative day 2. Two of these patients required revisional laparoscopic hemostasis surgery with hematoma evacuation and drainage. None of these patients developed a gastric staple line leak afterward (Table 2).

### Incisional hernia

An IH was found on follow-up clinical examination or abdominal CT scan in 65 patients (14.5%). More specifically, 31 patients (9.9%) in the LHC group and 34 patients (25%) in the TU group presented with an IH, with this difference reaching statistical significance (*P* < 0.0001). Thirty-six patients (55.4%) with symptomatic IH were treated by elective surgery with mesh placement, mostly in the TU group (23 patients (64%) (Table 2).

### Weight loss and resolution of comorbidities

The median percentage of total weight loss at 1 year was 30% [22.8–36] in the LHC group and 31.1% [25.9–35.6] in the TU group. The median percentage of EWL at 1 year was 84.4% [65.9–100.8] in the TU group and 72.9% [55.7–92.6] in the LHC group, with this difference reaching statistical significance (*P* = 0.0083). Overall, weight loss and comorbidities resolution at 1 year after SPSG were similar between the two groups. Weight loss and comorbidities evolution are depicted in Table 3.

**Table 3:** Weight loss and comorbidities evolution

Outcomes	LHC group (*n* = 313)	TU group (*n* = 136)	*P*
Weight loss, kg, median [IQR]	35 [25–45]	33 [28–39]	0.143
12 ;months post-surgery			
BMI reduction, kg/m^2^, median [IQR]	12.9 [10.1–17.5]	12.3 [10.4–14.5]	0.006
12 ;months post-surgery			
%TWL, median [IQR]	30 [22.8–36]	31.1[25.9–35.6]	0.698
12 ;months post-surgery			
%EWL, median [IQR]	72.9 [55.7–92.6]	84.4 [65.9–10.8]	0.008
12 ;months post-surgery			
Comorbidities resolution 12 ;months post-surgery	17 (51.5)	1 (20.0)	0.609
Diabetes resolution, *n* (%)	41 (55.4)	15 (75.0)	0.059
Hypertension resolution, *n* (%)	36 (75.0)	8 (88.9)	0.412
Dyslipidemia resolution, *n* (%)	117 (75.5)	20 (74.1)	0.761
OSAS resolution, *n* (%)	47 (57.3)	12 (31.5)	0.485
Fatty liver disease, *n* (%)	35 (25–45)	33 (28–39)	0.143

*LHC* left hypochondrium, *TU* transumbilical, *IQR* interquartile range, *TWL* total weight loss, *EWL* excess weight loss, *OSAS* obstructive sleep apnea syndrome

## Discussion

Over the years, our group has attained favorable outcomes by promoting and standardizing the SPSG technique, especially through the LHC approach. This approach is uniformly applied to all patients, regardless of comorbidities including those with superobesity [15], or previous abdominal surgery such as liver transplantation [16] and candidates for kidney transplantation [17]. It offers direct access to the surgical site, avoids digestive interposition, and ensures optimal alignment for stapling. This approach also facilitates standardization, aiding young surgeons who gain independence after approximately 20 procedures.

However, in response to the rising demand for minimally invasive cosmetic procedures, we have adopted the TU approach for selected patients. The TU access simplifies antral dissection and initial stapler application but presents challenges with dissection of the gastrosplenic ligament and final stapler applications due to distance and limited visual perspective. Overall, the LHC access is recommended for beginners and challenging cases, while TU access is suitable for select patients and experienced bariatric surgeons, but less conducive to routine practice.

Meticulous patient selection is crucial for achieving favorable outcomes in this technically demanding procedure. In this retrospective study, we observed that 93% of patients in the TU group were female, with lower BMI and fewer comorbidities. Existing literature reveals highly varied selection criteria. Maluenda et al. [10] recommended the TU approach for patients with a median BMI of 35 kg/m^2^. Lakdawala et al. [18] suggested it primarily for females with BMI less than 40 kg/m^2^, and Wang et al. [19] indicated feasibility even in patients with BMI ≥ 50 kg/m^2^. Our selection criterion is based on the distance between the left costal margin and the umbilicus, which is indirectly linked to BMI but also to gender, or more precisely to fat distribution. By using this precise selection criterion, we were able to carry out all the procedures via the TU approach without the need to add a trocar or convert to laparotomy, and without increasing the risk of intraoperative and early postoperative complications.

There was a 16-min longer operative duration in the LHC group. Several factors could explain this difference. Firstly, entry to the abdominal cavity at the umbilicus is easier due to the presence of finer subcutaneous tissue and a single-layer aponeurosis. Secondly, abdominal closure is faster in the umbilical cohort, whereas in the LHC cohort, closure is more complex due to the presence of a two-layer aponeurosis. However, the intraabdominal operative time was similar.

There was no difference in the length of stay between the two cohorts (3 days). Hospitalization and discharge follow an internal protocol, except for patients who have experienced complications. A fast-track protocol was recently implemented in our department, using a multimodal strategy to facilitate faster discharge.

This analysis demonstrates similar 1-year postoperative results between the two cohorts in terms of weight loss and resolution of comorbidities. This demonstrates that the TU access executed with adequate specific instruments in well-selected patients, does not result in the creation of a suboptimal sleeve that would impair long-term postoperative outcomes.

Since the early 2000s, SILS has become popular among many surgeons and is being applied to a broad range of bariatric and non-bariatric procedures. However, the rate of IH remains a major concern. A single but significant parietal incision might promote postoperative IH, particularly when the single-trocar is placed at the umbilicus, due to the inherent anatomical weakness of this area. The real incidence and prevalence of IH following bariatric surgery are not well defined in the literature. Some studies report a near 3% rate of IH following laparoscopic bariatric surgery [20], while others report rates as high as 24% and even 37% when detected by ultrasonography within 9 months after the bariatric procedure [12, 21, 22]. Several reasons can explain this heterogeneity: first, the focus on other postoperative outcomes, particularly weight loss. Second, clinical examination has proven to be unreliable for evaluating trocar-site hernias in bariatric patients, as most IH are not visible or palpable. Moreover, there’s a lack of long-term follow-up and the absence of imaging (ultrasound, CT scan) in the follow-up protocols in many bariatric centers. In a meta-analysis, Connel et al. [12] reported an increased risk of IH in those undergoing SILS (odds ratio 2.83). This is in line with results published by Antoniou et al. [23]. However, the majority of studies included in these meta-analyses [12, 23] focused solely on laparoscopic cholecystectomy, which may not be an ideal indication for this type of surgery. Indeed, the gallbladder can be removed in most cases through a 10- or 12-mm trocar incision during conventional laparoscopic cholecystectomy. Therefore, removing the specimen does not require making a single but large incision, as is done with SILS. In the present study, the global incisional hernia rate was 14% in the total cohort (65/449 patients) which is consistent with previous data reported by our group [24]. This rate may seem high to many surgeons but must be analyzed in context. Indeed, this result is comparable and equivalent to those from well-structured published studies [20, 22, 25] that have used specific imaging for the assessment of IH in conventional bariatric surgery. The most concerning aspect was the rate of IH (25%) in the TU group, whereas it was less 10% in the LHC cohort, even though surgery was performed in the latter group on patients with a higher BMI and more comorbidities. It’s interesting to note that 60% of patients who were scheduled for SPSG using the TU approach were found to have an umbilical hernia. More specifically, of the 82 patients who underwent TU surgery in the presence of an umbilical hernia, 25 developed an IH (30.5%), whereas of the 54 patients who underwent surgery without an umbilical hernia, only 9 developed an IH (16.7%). This difference was not statistically significant (*P* = 0.0732). However, it is conceivable that a certain number of small umbilical hernias were not detected during dissection and placement of the single-trocar which may overestimated the actual rate of postoperative IH in the “no umbilical hernia group.” Indeed, the prevalence of umbilical hernia in patients with obesity is notably higher compared to the general population. In a study published by Wang et al., the prevalence of umbilical hernia in adult surgically naive patients was around 65% based on CT findings [26]. After multivariate logistic regression analysis, age, asthma, and higher BMI were statistically the most significant risk factors [26]. The placement of preventive/curative mesh during TU approach seems imperative. Actually, current guidelines [27–29] for abdominal wall closure recommend the placement of prophylactic mesh during elective laparotomy closure in patients with obesity and clean or clean-contaminated operative fields. Systematic placement of a parietal prothesis in all patients with obesity operated by SILS is therefore probably necessary, given that this endoscopic surgery is in fact performed through a very small laparotomy incision. In our approach, the specimen (an intact lateral stomach) is removed through the single-port’s protective skirt which greatly reduces the risk of contamination of the operating field. We previously published a prospective study [24] (PRISM) suggesting that the prophylactic placement of a retromuscular mesh patch during SPSG through the LHC decreases the occurrence of postoperative IH and could be proposed for high-risk patients (BMI ≥ 45 kg/m^2^) without increasing the rate of postoperative surgical site infection. However, the placement of preventive mesh through the umbilicus in patients with obesity could be more challenging. The Intraperitoneal Onlay Mesh (IPOM) technique is an option, but the higher cost of bifacial mesh in potentially contaminated surgery settings could limit its use. The preperitoneal and retromuscular approaches allow placing a "low-cost" mesh. However, it requires a large dissection, which can be difficult in bariatric patients, particularly in the umbilicus. Onlay mesh placement might be an interesting strategy to strengthen the fascial closure. Based on the present results, we decided in the first instance to implant a parietal prothesis in all patients who presented with an umbilical hernia during surgical exploration and opted for IPOM which are more costly but relatively easy to fit. Depending on the results, we could discuss extending the indications to all patients operated using the TU approach.

This study has several limitations that should be highlighted. First of all, it was conducted by a team where SPSG is the standard for all patients undergoing sleeve gastrectomy. Therefore, the results cannot be easily generalized. Additionally, this study is subject to confounding bias due to its non-randomized methodology. Lastly, the overall benefit of SILS is debatable and needs to be demonstrated, especially considering the potential higher cost of preventive mesh placement. Improved cosmesis remains the primary benefit of the single-port approach for sleeve gastrectomy. Some may argue that such consideration is trivial in the context of bariatric surgery. However, esthetic improvement is often a significant concern in this population, particularly among young women, as evidenced by the increasing demand for SPSG in our department. Moreover, the psychological impact of this benefit may also contribute to enhanced patient quality of life and adherence to postoperative compliance. Large prospective multicenter studies are needed to evaluate the other potential benefits of SPSG, which have yet to be fully elucidated.

## Conclusion

In this retrospective study, we have demonstrated the feasibility of SPSG through two access routes: the LHC and TU approaches. Like any minimally invasive procedure, SPSG requires specific instrumentation and a dedicated learning curve. The LHC approach has been standardized and is routinely feasible for all patients eligible for LSG. Conversely, the TU access can be offered to selected patients. However, it is associated with a significantly much higher rate of IH. Prophylactic mesh placement could be considered to reduce the incidence of this complication. Our results should be validated by large, prospective, randomized studies, which should also evaluate the additional benefits of this type of procedure in bariatric surgery.
